# A comparison of ISO11140-1 specified type 4 versus type 5 internal chemical indicators for monitoring sterile packs in steam sterilization processes

**DOI:** 10.1017/ash.2025.10139

**Published:** 2025-09-03

**Authors:** Brian Kirk, Seto Wing Hong

## Abstract

**Objectives:** Six types of chemical indicators (CIs) are specified in ISO11140-1 for monitoring steam sterilization processes (SSP). Types3-6 are “internal” CIs, placed inside packs to assess attainment of sterilizing conditions. Type4 CIs have an ink patch which changes colour during the SSP. Type5 CIs exist as an ink patch which changes colour or a “moving front” (MF) format where a dye migrates along a wick reaching a pass/fail marker point. This study compares the requirements for type4 versus type5 CIs according to ISO11140-1, evaluates the claims of some type4 and 5 CIs with regard SSP parameters, and tests the ability of type5 CIs to meet specified performance and variability of response. **Methods:** We reviewed ISO requirements and stated commercial claims of type4 and 5 CIs. Samples of several Type5 CIs were exposed to increasing times at a temperature of 121^o^C in a CI Evaluating Resistometer. Upon completion of the test cycle samples were removed and the length of the ink migration measured and charted. **Results and Conclusions:** According to ISO11140-1 requirements, Type4 CIs need not respond to all SSP variables. Type4 CIs can have stated values (SV) which are unrelated to SSP parameters used. The tolerances for the pass/fail window for a Type5 CI is better than for a Type4. The performance of a Type5 CI has a relationship to that of a biological indicator (BI). The performance requirements for Type5 CIs are more prescriptive. MF- CIs provide greater clarity for interpretation compared to the colour change of some CIs. The SVs and colour change of type4 CIs are specified by the manufacturer. The minimum SVs for Type5 CIs are prescribed in ISO11140-1. When tested, there was considerable variation in the response of some Type5 MF-CIs; a factor not addressed in ISO11140-1.

